# Metabolomics study of fasudil on cisplatin-induced kidney injury

**DOI:** 10.1042/BSR20192940

**Published:** 2019-11-19

**Authors:** Demeng Xia, Xueli Lai, Kaiwen Wu, Panyu Zhou, Lei Li, Zhiyong Guo, Shuogui Xu

**Affiliations:** 1 Department of Emergency, Shanghai Changhai Hospital, No.168, Changhai St, Shanghai 200433, PR China; 2 Department of Nephrology, Shanghai Changhai Hospital, No.168, Changhai St, Shanghai 200433, PR China; 3 Department of Ultrasound, Shanghai Pulmonary Hospital, Affiliated to Tongji University School of Medicine, No.503, Zhengmin St Shanghai 200433, PR China

**Keywords:** cisplatin-induced kidney injury, fasudil, metabolomics

## Abstract

Fasudil is a derivative of 5-isoquinoline sulfonamide, which is a Rho kinase inhibitor, a wide range of pharmacological effects. Fasudil has been shown to attenuate kidney injury caused by certain substances. In the present study, metabolomic analysis of mouse kidney tissues ultra-performance liquid chromatography/quadrupole time-of-flight mass spectrometry was used to determine the metabolomic changes in cisplatin-induced kidney injury and the fasudil-induced attenuation of cisplatin-induced kidney injury. Metabolomic profiling of kidney tissues revealed significant differences in metabolites between the control group and the cisplatin group and between the cisplatin group and the fasudil-intervention group. With metabolomic approach, 68 endogenous differential metabolites were found, and multivariate statistical analysis, accurate molecular weights, isotope tracers, mass-spectrometry secondary-fragment information, and standard-reference comparisons were used to identify these substances. Based on these differential metabolites, a metabolic-pathway network was constructed and revealed that fasudil primarily attenuated cisplatin-induced renal injury by modulating lipid and amino-acid metabolism. These results further demonstrate that kidney injury can be induced by cisplatin and, moreover, suggest that fasudil can be used to reduce kidney injury at early stages in patients treated with cisplatin.

## Introduction

Cisplatin (CDDP) is a platinum-based chemotherapy drug that inhibits cellular division and exhibits broad-spectrum anti-tumor activity. CDDP can be used to treat solid tumors, such as those caused by head and neck cancer, testicular cancer, ovarian cancer, and lung cancer [1]. Cisplatin can bind to DNA bases and interfere with DNA synthesis to kill cells [2]. However, its side effects—which include severe ototoxicity, nephrotoxicity, and myelosuppression [3]—limit the use of CDDP [4]. Nephrotoxicity is the primary adverse reaction of CDDP. The increase in risk factors for nephrotoxicity can be related to the dose and frequency of CDDP use, as well as factors such as female sex, old age, and smoking. The most important and severe effect of CDDP in the kidney is acute kidney injury (AKI), and the incidence of AKI caused by CDDP is relatively high [5]. CDDP-induced kidney injury is a complex process involving multiple pathways that can cause changes at the molecular level [6,7].

AKI refers to acute injury to kidney function, including acute mild renal dysfunction [8]. Two main phenomena highlight the severity of AKI. First, AKI has serious consequences, and repeated AKI can induce chronic kidney disease [4]. For some critically ill patients, AKI is considerably dangerous and the mortality rate has been reported to be approximately 60% in such cases. [9]. Second, the incidence of AKI is markedly high, reaching 1–7% in inpatients and 1–25% in intensive-care-unit patients [10]. Additionally, the incidence of AKI is increasing over the years [11]. Efforts toward the prevention, early diagnosis, and early treatment of AKI have therefore drawn extensive attention. In recent years, several AKI markers have been discovered, including kidney injury molecule-1 (KIM-1), neutrophil gelatinase associated lipocalin (NGAL), fibroblast growth factor-23 (FGF-23), tissue inhibitor of metalloproteinase-2 (TIMP-2), insulin-like growth factor binding protein 7 (IGFBP7), cystatin c (Cys-C), and cysteine-rich 61 (CYR-61) [12,13]. The kidney is the main target of drug toxicity, so drug-induced AKI is common in clinical practice. The severe nephrotoxicity associated with CDDP can cause AKI [14]. Identifying potential markers of CDDP-induced kidney injury and understanding the mechanisms concomitant damage may facilitate earlier diagnosis and intervention of AKI.

Fasudil (Fas) is a widely used Rho enzyme inhibitor that inhibits a variety of protein kinases and is currently used as a novel cerebrovascular dilator [15]. Studies have shown that Rho enzyme can cause renal tubule damage [16]. Fas, a selective Rho enzyme inhibitor, has been shown to have protective effects on renal fibrosis [17,18], and Fas can inhibit epithelial–myofibroblast transdifferentiation of renal tubular epithelial cells due to high sugar differentiation of renal tubular epithelial fibroblasts [19,20]. Fas can also mitigate the progression of chronic renal failure, and its specific mechanisms have been gradually elucidated [21]. In addition, Fas can alleviate kidney injury caused by some drugs. For example, Fas can attenuate nephrotoxicity induced by cyclosporine and daunorubicin [17,22].

Metabolomics, an outgrowth of genomics and proteomics, is an emerging systematic biological method that comprehensively explores the numerous metabolites in complex biological systems after pathological stimulations and drug treatments [13,23]. In this way, metabolomics is a powerful means of exploring potential biomarkers for diagnosis, treatment, and prognosis of various diseases [24–26]. Kidney injury is a complex pathophysiological process, and metabolomics has helped establish its pathological mechanism from the level of endogenous metabolites and has been used in many research applications [27,28]. In the present study, metabolic analysis of kidney tissues was performed to investigate changes in the metabolic regulatory network in a model of CDDP-induced kidney injury, and the biomarkers and pathophysiological mechanisms of CDDP-induced kidney injury were explored. Additionally, the therapeutic effects and mechanisms of Fas on CDDP-induced kidney injury were evaluated.

## Materials and methods

### Chemicals and reagents

CDDP was purchased from Sigma–Aldrich Co. Ltd. (St. Louis, MO, U.S.A.). Fas was purchased from Chase Sun Co., Ltd. (Tianjin, China), high-performance liquid chromatography (HPLC) grade methanol and acetonitrile were purchased from Merck & Co. (Darmstadt, Germany), formic acid was purchased from Fluka Chemicals Ltd. (Buchs, Switzerland), ultrapure water was prepared from the Milli-Q ultrapure water system (Millipore, Bedford, MA, U.S.A.) in our lab, and other reagents were of analytical grade. An F4/80 antibody was purchased from Cell Signaling Technology (Boston, U.S.A.)

### Experimental equipment

The following experimental instruments were used: an Agilent 1290 Infinity liquid chromatography (LC) system (US); an Agilent 6538 Accurate-Mass Q-TOFMS quadrupole-time-of-flight tandem mass spectrometer (with electrospray ion source) (U.S.A.); and a Thermo Fisher PRESCO 17 high-speed refrigerated centrifuge.

### Experimental animals

Specific pathogen-free (SPF) wild-type C57BL/6 mice (20–25 g, male, 7–8 weeks old) were purchased from Shanghai SLAC Laboratory Animals Co., Ltd. There were a total of 24 mice (distributed into the following groups: six mice in the control group (NS); six mice in the CDDP-induced AKI model group (CDDP); six mice in the high-dose drug-intervention group (CDDP+High Fas); and six mice in the low-dose drug intervention group (CDDP+Low Fas). All mice were housed in a room with humidity and temperature controlled at approximately 60% and 22 ± 2°C, respectively, with a 12-hour light/dark cycle. Normal animal feed and distilled water were provided before administration of drugs. The mice were allowed to adapt to the new environment and were then randomly divided into the four groups. The mice in the e CDDP+High Fas group were given 40 mg/kg of Fas via intraperitoneal injection, and the mice in the CDDP+Low Fas group were given 20 mg/kg of Fas via intraperitoneal injections. Two days later, 20 mg/kg of CDDP was intraperitoneally injected into the mice of the CDDP group and drug intervention groups, and the same volume of normal saline was intraperitoneally injected into the mice in the NS group. The injections were performed for three days (once a day). Animal experiment was carried out at the Animal Experimental Center of the Second Military Medical University and the animal experiments described above were reviewed and approved by the Medical Research Ethics Committee of the Changhai Hospital (Approval number: No. SCXK 2018-0001).

### Sample collection and processing

#### Sample collection

The NS group, CDDP group, CDDP+High Fas group, and CDDP+Low Fas group were subjected to blood collection through the orbital venous plexus at 24 h after the last drug/vehicle administration. In order to determine the optimal dose of fasudil, the biochemical indicators and serum kidney injury molecule-1 (KIM-1) were detected. The mice were killed by cervical dislocation. After the mice were subjected to heart perfusion, and bilateral kidneys were collected. The kidney from one side was subjected to conventional hematoxylin–eosin staining and immunohistochemical staining of F4/80. The degree of injury in renal tissue was observed under light microscopy. The proportions of different types of kidney injuries—specifically swelling of renal tubular epithelial cells, vacuolar degeneration, necrosis, and desquamation—were graded and the semi-quantitative scores of the injuries were determined as follows: 0: no damage; 1: 10%; 2: 11–25%; 3: 26–45%; 4: 46–75%; and 5: >75% [29]. Additionally, a number of F4/80-positive cells were observed and quantified under light microscopy. The kidney from the other side of the body of each mouse was stored in a refrigerator at −80°C for the subsequent metabolomic analysis.

#### Sample pretreatments

The kidney tissues were thawed in a refrigerator at 4°C, and 100 μl of kidney was transferred into a 1.5-ml centrifuge tube, followed by addition of 300 μl of methanol. The mixture was vortexed for 5 min, centrifuged at 13,000 rpm for 4 min at 4°C, and allowed to stand at 4°C for 10 min. The supernatant was collected for ultra-performance liquid chromatography-tandem mass spectrometry (UPLC-MS) analysis. The QC samples were prepared by pooling aliquots from all kidney tissue samples (NS, CDDP, CDDP+High Fas groups) collected in the course of the study.

### Examination of biochemical indicators

The collected blood samples of the mice were examined in the laboratory, and the kidney function of each mouse was determined by assessing the contents of creatinine and urea nitrogen (BUN) in the blood.

### Detection of serum kidney injury molecule-1 (KIM-1)

The collected blood samples of the mice were subjected to laboratory examination, and KIM-1 levels were determined using an enzyme-linked immunosorbent assay (ELISA) kit (Uscn, Wuhan, China) according to the manufacturer’s instructions.

### UPLC-MS analysis

#### Chromatographic conditions

Chromatographic separation was performed using an ACQUITY UPLC HSS T3 column (2.1 mm × 100 mm, 1.8 μm, Waters, Milford, MA, U.S.A.). Reverse phase (RP) liquid chromatography and hydrophilic interaction (HILIC) liquid chromatography were performed as follows: mobile phase A was 0.1% aqueous formic acid, and mobile phase B was acetonitrile (0.1% formic acid). Gradient elution was used, and the gradient program was set as follows: 0–2 min, 5% B; 2–13 min, 5–95% B; and 13–15 min, 95% B. The column was equilibrated for 5 min, the column temperature was 40°C, the flow rate was 400 μl/min, and the injection volume was 4 μl. HILIC conditions: Gradient elution was used, and the gradient program was set as follows: 95%B at 0–5 min, 95–87%B at 5–6 min, 87–82%B at 6–12 min, 82–75%B at 12–15 min. The column was equilibrated for 5 min, the column temperature was 40°C, the flow rate was 400 μl/min, and the injection volume was 4 μl.

#### Operating conditions for mass spectrometry

Mass spectrometry analysis was performed using an electrospray ion source in positive and negative ion modes. Positive ion mode parameters were as follows: capillary voltage, 4000 V; drying gas flow rate, 11 l/min; drying gas temperature, 350°C; nebulizing gas pressure, 45 psig, fragmentor voltage, 120 V; skimmer voltage 60 V, data acquisition range *m/z* 100–1100; the internal standard ions of *m/z* 121.0509 and *m/z* 922.0098 were selected for real-time mass calibration. Negative ion mode parameters were as follows: capillary voltage, 3500 V; drying gas flow rate,11 l/min;dry gas temperature, 350°C; nebulizing gas pressure, 45 psig; fragmentor voltage 120 V, skimmer voltage 60 V; and data acquisition range *m/z* 100–1100; The internal standard ions of *m/z* 112.985587 and *m/z* 1033.988109 were selected for real-time mass calibration. The potential biomarker ions were further subjected to MS/MS analysis and the collision energy was adjusted between 10 and 50 V depending on the ionic conditions. The QC samples were randomly inserted in the sequence to validate the stability of the system. PCA was employed to assess the clustering of the QC samples in the PCA score plot from all the tested samples in HILIC and RP.

### Data analysis

#### Data preprocessing

Raw data were converted to a universal format via Agilent MassHunter Qualitative software prior to pattern recognition. The converted data were further subjected to peak calibration and peak integration by XCMS (http://metlin.scripps.edu/download/). Finally, a 3D data matrix of retention time, mass-to-charge ratio, and peak intensity was produced. The modified 80% rule was used to remove missing values (i.e. to remove MS ions with a frequency [non-zero value] below 80% in a certain group). Data were centralized and normalized using MATLAB.

#### Statistical analysis

One-way analysis of variance (ANOVA) was performed using SPSS 11.0 software (IBM). The statistically significant differences among the three groups were compared. A *P* < 0.05 was considered indicative of statistical significance. Centralized and normalized data were imported into SIMCA-P V11.0 (Umetrics, Sweden) for principal component analysis (PCA) and partial least squares-discriminant analysis (PLS-DA), and the model was evaluated based on related *R*^2^ and *Q*^2^ values.

## Results

### Biochemical and histological measurement

**Figure 1:**
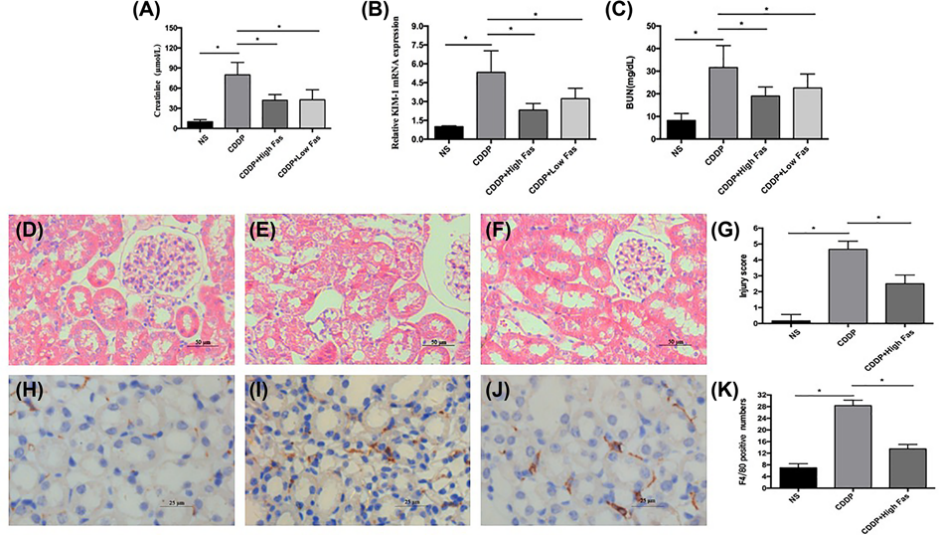
Changes in serum creatinine KIM-1, and BUN in the NS, CDDP, CDDP+ High Fas, and CDDP+ Low Fas Groups (**A–C**); HE staining of kidney tissues (**D–G**), F4/80-positive cell counts in kidney tissues (**H–K**) in the NS, CDDP, and CDDP+ High Fas Groups. Serum creatinine, KIM-1, and BUN levels in CDDP group were significantly higher than those in the NS group, CDDP+High Fas, and CDDP+Low Fas groups were lower than those in the CDDP group. We chose a high dose of Fas as the intervention group in subsequent studies. Compared with that in the NS group, the renal tubular structure in the CDDP group was disordered and vacuolated, indicating that the kidney injury was severe, while the renal injury in the CDDP+High Fas group was alleviated, and semi-quantitative scores of renal injury and counts of F4/80 positive cells also reflected the same trend (* statistically significant, *P* < 0.05) .

To confirmed the successful construction of mice models that showed the nephrotoxicity of cisplatin and screened the optimally protective dosage of the fasudil. We detected the two indicators commonly used in renal function: creatinine and urea nitrogen (BUN), and the level of AKI markers: the serum kidney injury molecule 1 (KIM-1). Just as shown in Figure 1A–C, Serum creatinine, KIM-1, and BUN levels in CDDP group were significantly higher than NS group, which indicated the kidney injury of nephrotoxicity. In addition, CDDP+High Fas and CDDP+Low Fas groups were lower than CDDP group, which proved the intervention of the fasudil. Although, there were no statistical difference between CDDP+High Fas and CDDP+Low Fas groups, CDDP+High Fas group had a lower trend. In thus, a high dose of fasudil at 40 mg/kg was defined as the optimal dose for the follow-up drug intervention group and used in next histological measurement and metabonomics analysis. Just as shown in Figure 1D–G, compared with the NS group, the renal tubular structure in the CDDP group was disordered and vacuolated, indicating that the kidney injury was severe, while the renal injury in the CDDP+High Fas group was alleviated, and semi-quantitative scores of renal tissue injury reflect the same trend (Figure 1H-K). It has been reported that inflammation plays a major role in the pathophysiology of AKI [30]. Meanwhile, inflammation was evaluated by protein expression of the F4/80 macrophage marker, the counts showed the same trend, which further confirmed CDDP-induced kidney injury and the protective effects of Fas.

### Metabolomics profiling

**Figure 2:**
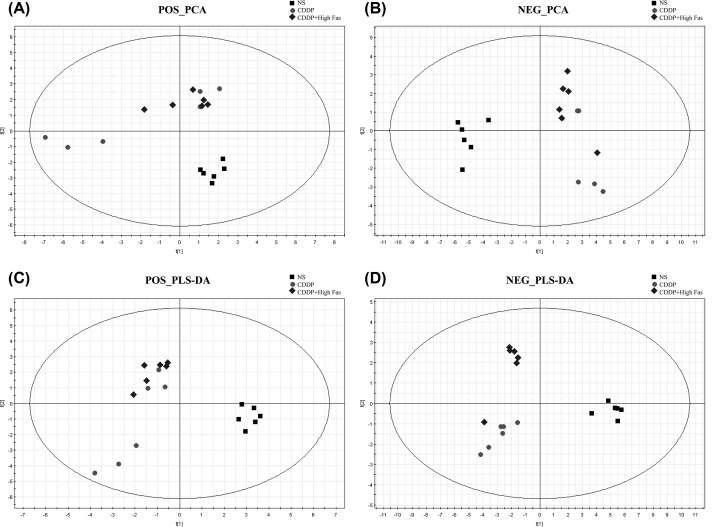
PCA score plots (**A** and **B**) and PLS-DA (**C** and **D**) score plots of kidney tissue samples in the NS, CDDP, and CDDP+ High Fas Groups across different time points via HILIC-MS methods in positive and negative modes The samples from different groups showed differences in the PCA score plots and PLS-DA score plots from the NS, CDDP, and CDDP+ High Fas Groups were clustered together and were clearly separated.

**Figure 3:**
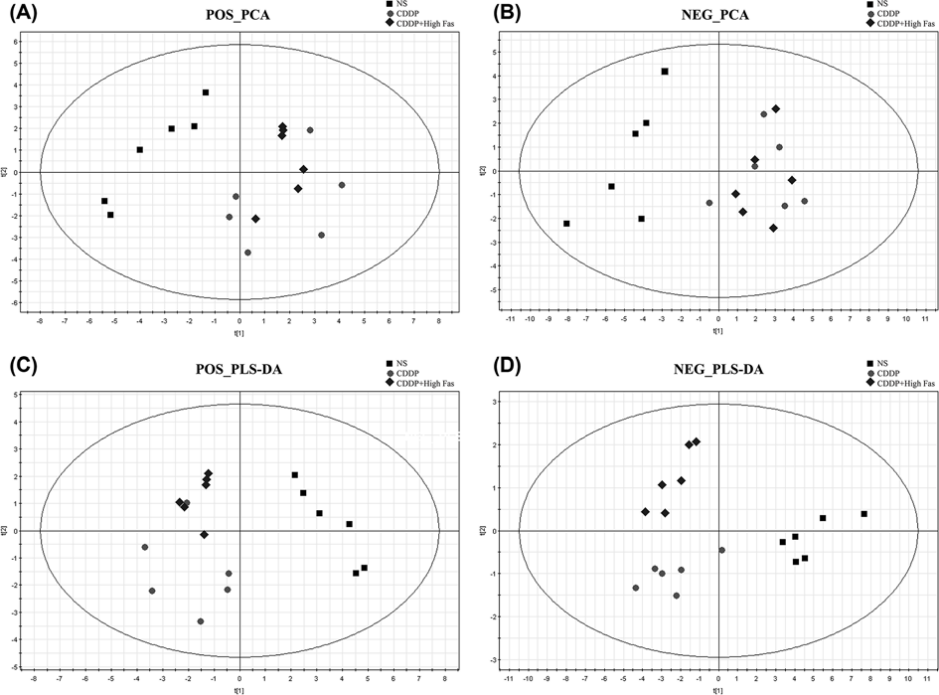
PCA score plots (**A** and **B**) and PLS-DA (**C** and **D**) score plots of kidney tissue samples in the NS, CDDP, and CDDP+ High Fas Groups across different time points via RPLC-MS methods in positive and negative modes The samples from different groups showed differences in PCA score plots and PLS-DA score plots from the NS, CDDP, and CDDP+ High Fas Groups clustered together and were clearly separated.

PCA score plot including all the test and QC samples shows that the QC sample features were tightly clustered in Supplementary Figures S1 and S2. The results demonstrated that the stability of the proposed method was satisfying. According to previous UPLC-MS conditions, kidney tissues samples of the mice from the NS group, CDDP group, and the CDDP+High Fas group were analyzed in hydrophilic interaction liquid chromatography (HILIC) and Reversed-phase chromatography (RPLC) modes. To investigate the CDDP-induced nephrotoxicity and changes in urinary metabolic profiles in mice following Fas intervention, unsupervised PCA and PLS-DA was used to determine differences in metabolites among the three groups, as shown in Figures 2 and 3 in different modes. There was a clear separation trend in the NS group, CDDP group, and CDDP+Fas group, indicating a certain degree of difference among the three groups. In HILIC modes, when three components were calculated in the positive mode, the cumulative R^2^X, R^2^Y, and Q^2^ were 0.432, 0.975, and 0.487, respectively, while the cumulative R^2^X, R^2^Y, and Q^2^ in the negative mode were 0.773, 0.946, and 0.684. Meanwhile, the cumulative R^2^X, R^2^Y, and Q^2^ were 0.612, 0.965, and 0.592 in RPLC positive modes and 0.587, 0.957, and 0.415 in RPLC negative modes. No over-fitting was observed in either ESI positive or ESI negative according to the results of the permutation test.

### Screening potential biomarkers

**Figure 4:**
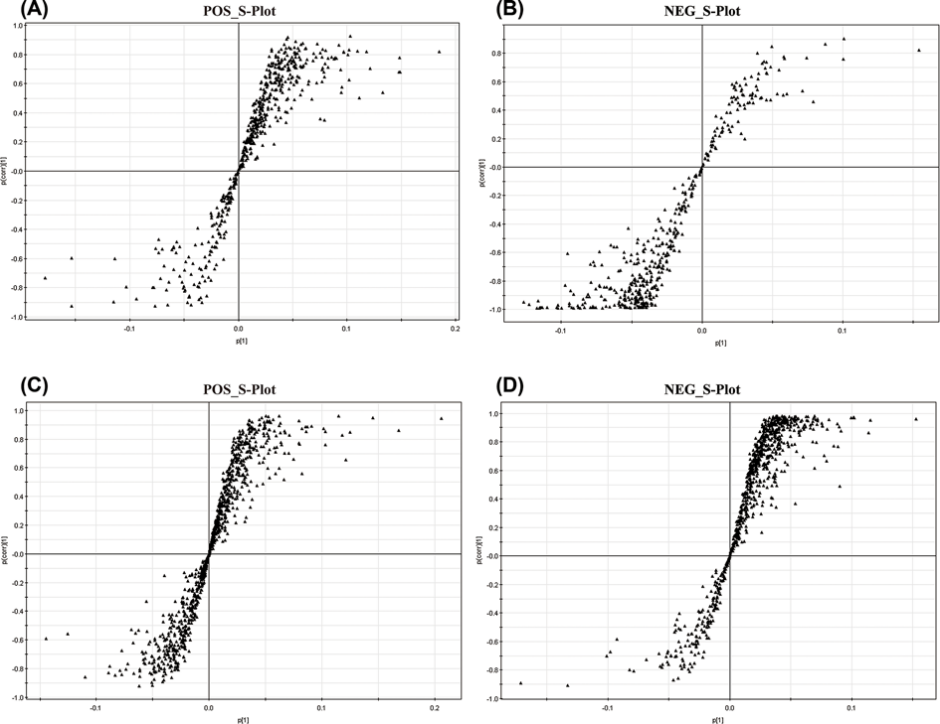
The S-plot of kidney tissue samples in the NS, CDDP, and CDDP+ High Fas Groups across different time points via HILIC-MS (**A** and **B**) and RPLC-MS (**C** and**D**) methods in positive and negative modes The samples from different groups showed significant differences. Each point in the S-plot represents an ion. Ions far away from the origin were significantly important to the differences among the three groups and had greater VIP values.

To identify biomarkers associated with CDDP-induced nephrotoxicity and the renal protection effect of Fas, we used a PLS-DA method to produce the score plot and scatter plot (S-Plot) of the PLS-DA for both HILIC and RPLC modes in the positive and negative ion modes. As shown in Figure 4, there was an apparent separation among the NS group, CDDP group, and CDDP+ High Fas group. In the S-plot, a greater distance from the origin indicated a greater contribution to the difference among the groups. In this experiment, we considered metabolites with a variable importance in projection (VIP) > 1.0 to be representative of differential metabolites. We further compared the differential variables among the NS group, CDDP group, and CDDP+ High Fas group using one-way ANOVA. The variables that differed significantly among the three groups were considered differential variables. A preliminary assignment of the mass-spectral peaks was performed based on the element-matching function provided by high-resolution mass spectrometry data, as well as via Metlin (http://metlin.scripps.edu/), HMDB (http://www.hmdb.ca/), and Mass Bank (http://www.massbank. The jp/) database searches. Further high-resolution MS/MS analysis of the samples was performed to obtain mass-spectrometry fragment information of differential metabolites, and the differential metabolites were structurally confirmed by network database searches and comparisons with standards. Finally, 68 differential metabolites associated with the cisplatin nephrotoxicity and the kidney protection of fasudil were identified, shown in Table 1. The results indicate that the metabolic pattern in the kidney of mice with cisplatin-induced renal injury was reversed to normal levels after fasudil treatment.

**Table 1:** Potential biomarkers related to protective effects of fasudil in cisplatin induced nephrotoxicity

No.	*R*_t_/min	Quasi-molecularion (*m/z*)^1^			Error (ppm)	Model	Formula	Metabolites	Fold change		*P* value^4^
		Observed	Theoretical	Ion					CDDP/NS	CDDP +Fas/CDDP	
1	6.58	782.569	781.562	[M+H]+	0	HILIC^2^	C_44_H_80_NO_8_P	PC(20:3/16:1)	0.257	2.583	0.002
	14.69	826.560	781.562	[M+FA-H]-	0	RP^3^			0.543	1.157	0.020
2	5.63	850.561	805.562	[M+FA-H]-	0	HILIC	C_46_H_80_NO_8_P	PC(20:4/18:2)	0.735	1.889	0.005
3	5.55	854.591	809.594	[M+FA-H]-	0	HILIC	C_46_H_84_NO_8_P	PC(20:4/18:0)	0.447	1.690	0.004
4	5.86	824.545	779.547	[M+FA-H]-	0	HILIC	C_44_H_78_NO_8_P	PC(20:4/16:1)	0.237	1.873	0.000
	13.14	824.545	779.547	[M+FA-H]-	0	RP			0.294	0.984	0.000
5	2.52	716.522	715.515	[M+H]+	0	HILIC	C_39_H_74_NO_8_P	PE(16:1/18:1)	2.414	1.575	0.007
6	2.33	762.505	739.515	[M+Na]+	0	HILIC	C_41_H_74_NO_8_P	PE(20:3/16:1)	2.496	2.773	0.001
	14.94	738.508	739.515	[M-H]-	0	RP			1.701	1.394	0.035
7	2.27	790.537	789.531	[M+H]+	1	HILIC	C_45_H_76_NO_8_P	PE(20:4/20:3)	3.319	2.187	0.000
	2.22	788.523	789.531	[M-H]-	0	HILIC			1.115	1.920	0.009
8	2.29	762.508	763.515	[M-H]-	0	HILIC	C_43_H_74_NO_8_P	PE(20:4/18:2)	1.784	2.000	0.000
9	1.62	885.551	886.557	[M-H]-	1	HILIC	C_47_H_83_O_13_P	PI(20:4/18:0)	3.003	0.561	0.012
10	10.06	775.597	730.599	[M+FA-H]-	0	HILIC	C_41_H_83_N_2_O_6_P	SM(d18:1/18:0)	2.218	1.523	0.006
11	11.49	546.354	545.348	[M+H]+	2	HILIC	C_28_H_52_NO_7_P	LysoPC(20:3)	0.323	1.099	0.004
	10.74	546.355	545.348	[M+H]+	0	RP			0.176	0.764	0.000
	10.73	590.347	545.348	[M+FA-H]-	1	RP			0.133	0.828	0.000
12	11.45	566.321	543.333	[M+Na]+	1	HILIC	C_28_H_50_NO_7_P	LysoPC(20:4)	0.182	1.042	0.000
	11.46	544.339	543.333	[M+H]+	1	HILIC			0.269	1.092	0.000
	10.24	588.332	543.333	[M+FA-H]-	2	RP			0.435	1.112	0.031
13	11.18	568.363	523.364	[M+FA-H]-	1	HILIC	C_26_H_54_NO_7_P	LysoPC(18:0)	0.452	1.007	0.000
14	11.53	522.355	521.348	[M+H]+	0	HILIC	C_26_H_52_NO_7_P	LysoPC(18:1)	0.471	1.002	0.000
	11.07	522.354	521.348	[M+H]+	2	RP			0.594	0.913	0.005
	11.07	544.337	521.348	[M+Na]+	0	RP			0.687	0.912	0.004
	11.06	566.346	521.348	[M+FA-H]-	0	RP			0.435	1.047	0.000
15	11.65	542.322	519.333	[M+Na]+	0	HILIC	C_26_H_50_NO_7_P	LysoPC(18:2)	0.219	1.163	0.000
	11.61	520.340	519.333	[M+H]+	0	HILIC			0.185	1.226	0.000
	10.34	520.338	519.333	[M+H]+	3	RP			0.484	0.954	0.000
16	11.69	518.322	495.333	[M+Na]+	0	HILIC	C_24_H_50_NO_7_P	LysoPC(16:0)	0.568	1.092	0.023
	10.68	540.330	495.333	[M+FA-H]-	1	RP			0.649	0.620	0.023
17	9.99	494.324	493.317	[M+H]+	0	RP	C_24_H_48_NO_7_P	LysoPC(16:1)	0.534	0.981	0.001
	9.97	538.316	493.317	[M+FA-H]-	1	RP			0.380	1.106	0.000
18	10.19	476.279	477.286	[M-H]-	1	RP	C_23_H_44_NO_7_P	LysoPE(18:2/0:0)	0.350	1.018	0.003
19	11.45	433.236	434.243	[M-H]-	0	RP	C_21_H_39_O_7_P	LysoPA(18:2/0:0)	0.461	1.780	0.001
20	10.76	368.257	367.249	[M+H]+	2	RP	C_17_H_38_NO_5_P	Sphinganine 1-phosphate	2.536	1.148	0.001
21	9.58	380.255	379.249	[M+H]+	2	RP	C_18_H_38_NO_5_P	Sphingosine 1-phosphate	0.733	0.724	0.012
	9.56	378.242	379.249	[M-H]-	1	RP			0.489	0.806	0.000
22	0.67	140.068	117.079	[M+Na]+	1	RP	C_5_H_11_NO_2_	L-Valine	2.625	0.769	0.013
23	23.79	147.113	146.106	[M+H]+	1	HILIC	C_6_H_14_N_2_O_2_	L-Lysine	1.168	0.666	0.006
24	7.87	116.071	115.063	[M+H]+	3	HILIC	C_5_H_9_NO_2_	L-Proline	0.908	0.663	0.000
25	2.89	205.097	204.090	[M+H]+	0	RP	C_11_H_12_N_2_O_2_	L-Tryptophan	0.379	0.777	0.001
	2.91	203.082	204.090	[M-H]-	2	RP			0.263	0.920	0.000
26	2.90	188.070	165.079	[M+Na]+	9	RP	C_9_H_11_NO_2_	L-Phenylalanine	0.390	0.789	0.001
27	0.92	182.080	181.074	[M+H]+	6	RP	C_9_H_11_NO_3_	L-Tyrosine	0.544	0.967	0.001
28	0.69	136.044	135.035	[M+H]+	9	RP	C_4_H_9_NO_2_S	L-Homocysteine	3.101	0.908	0.000
29	1.49	209.091	208.085	[M+H]+	5	RP	C_10_H_12_N_2_O_3_	L-Kynurenine	1.989	0.757	0.000
30	18.04	162.112	161.105	[M+H]+	2	HILIC	C_7_H_15_NO_3_	L-Carnitine	0.434	0.810	0.000
31	15.13	218.139	217.131	[M+H]+	1	HILIC	C_10_H_19_NO_4_	Propionylcarnitine	0.328	1.038	0.006
	1.09	218.138	217.131	[M+H]+	3	RP			0.268	1.392	0.020
32	10.75	400.342	399.335	[M+H]+	0	HILIC	C_23_H_45_NO_4_	Palmitoylcarnitine	2.020	0.837	0.000
	10.60	400.342	399.335	[M+H]+	0	RP			2.569	0.823	0.000
33	10.48	426.357	425.351	[M+H]+	1	HILIC	C_25_H_47_NO_4_	Vaccenylcarnitine	2.158	1.086	0.012
	10.76	426.357	425.351	[M+H]+	1	RP			3.014	0.792	0.004
34	10.23	424.341	423.335	[M+H]+	2	RP	C_25_H_45_NO_4_	Linoleylcarnitine	2.135	0.958	0.006
35	11.36	428.373	427.366	[M+H]+	1	RP	C_25_H_49_NO_4_	Stearoylcarnitine	2.860	0.728	0.017
36	10.90	343.224	320.235	[M+Na]+	1	RP	C_20_H_32_O_3_	16R-HETE	1.696	0.626	0.043
37	0.84	303.234	304.240	[M-H]-	3	HILIC	C_20_H_32_O_2_	Arachidonic Acid	0.320	0.983	0.000
38	0.84	279.234	280.240	[M-H]-	3	HILIC	C_18_H_32_O_2_	Linoleic acid	0.542	1.288	0.006
39	0.68	203.052	180.063	[M+Na]+	2	RP	C_6_H_12_O_6_	Glucose	2.186	0.858	0.000
40	0.70	195.051	196.058	[M-H]-	0	RP	C_6_H_12_O_7_	Gluconic acid	7.019	0.713	0.011
41	1.56	87.009	88.016	[M-H]-	2	HILIC	C_3_H_4_O_3_	Pyruvic acid	0.400	1.173	0.001
42	0.68	148.003	125.015	[M+Na]+	5	RP	C_2_H_7_NO_3_S	Taurine	2.116	0.900	0.025
	0.72	126.022	125.015	[M+H]+	0	RP			2.420	0.939	0.016
43	0.82	215.015	192.027	[M+Na]+	5	RP	C_6_H_8_O_7_	Citric acid	5.094	0.695	0.001
	0.82	191.019	192.027	[M-H]-	3	RP			2.245	1.085	0.040
44	0.83	173.009	174.016	[M-H]-	0	RP	C_6_H_6_O_6_	cis-Aconitic acid	3.343	0.662	0.012
45	1.00	117.019	118.027	[M-H]-	2	RP	C_4_H_6_O_4_	Succinic acid	0.212	1.347	0.003
46	1.17	179.035	180.042	[M-H]-	0	HILIC	C_9_H_8_O_4_	Caffeic acid	0.476	2.631	0.049
47	1.85	220.118	219.111	[M+H]+	0	RP	C_9_H_17_NO_5_	Pantothenic acid	3.464	1.307	0.001
	1.86	218.103	219.111	[M-H]-	1	RP			2.277	1.452	0.001
48	0.83	129.020	130.027	[M-H]-	5	RP	C_5_H_6_O_4_	Mesaconic acid	2.768	0.810	0.013
49	5.30	206.081	205.074	[M+H]+	0	RP	C_11_H_11_NO_3_	Indolelactic acid	3.615	1.017	0.002
	5.30	204.066	205.074	[M-H]-	3	RP			2.772	1.221	0.008
50	0.92	129.016	106.027	[M+Na]+	1	RP	C_3_H_6_O_4_	Glyceric acid	4.132	0.667	0.002
51	1.01	127.037	104.047	[M+Na]+	3	RP	C_4_H_8_O_3_	3-Hydroxybutyric acid	3.080	0.760	0.010
52	1.55	267.073	268.081	[M-H]-	1	HILIC	C_10_H_12_N_4_O_5_	Inosine	0.384	1.006	0.000
53	12.19	132.076	131.070	[M+H]+	5	HILIC	C_4_H_9_N_3_O_2_	Creatine	2.950	0.778	0.026
54	2.29	114.066	113.059	[M+H]+	1	HILIC	C_4_H_7_N_3_O	Creatinine	5.343	0.731	0.014
55	1.26	61.040	60.032	[M+H]+	5	HILIC	CH_4_N_2_O	Urea	3.544	0.796	0.008
56	3.28	167.021	168.028	[M-H]-	0	HILIC	C_5_H_4_N_4_O_3_	Uric acid	0.463	1.351	0.006
	0.81	167.021	168.028	[M-H]-	0	RP			0.443	1.509	0.000
57	0.68	154.048	131.058	[M+Na]+	3	RP	C_5_H_9_NO_3_	N-Acetyl-alanine	3.795	1.133	0.000
58	24.73	189.160	188.153	[M+H]+	1	HILIC	C_9_H_20_N_2_O_2_	Trimethyllysine	3.519	0.673	0.019
59	0.91	189.123	188.116	[M+H]+	1	RP	C_8_H_16_N_2_O_3_	N-acetyllysine	7.078	0.527	0.004
60	2.97	192.067	193.074	[M-H]-	1	HILIC	C_10_H_11_NO_3_	Phenylacetylglycine	3.754	1.414	0.000
	4.54	194.081	193.074	[M+H]+	0	RP			8.340	1.014	0.000
	4.55	192.067	193.074	[M-H]-	1	RP			4.908	1.224	0.000
61	6.97	137.071	136.064	[M+H]+	0	HILIC	C_7_H_8_N_2_O	Aminobenzamide	12.142	1.133	0.009
62	1.03	197.032	152.033	[M+FA-H]-	1	HILIC	C_5_H_4_N_4_O_2_	Xanthine	0.375	1.473	0.000
63	1.12	181.033	158.044	[M+Na]+	1	HILIC	C_4_H_6_N_4_O_3_	Allantoin	5.820	0.504	0.015
	0.71	157.036	158.044	[M-H]-	4	RP			2.195	0.805	0.002
64	0.67	212.003	213.010	[M-H]-	3	HILIC	C_8_H_7_NO_4_S	Indoxyl sulfate	9.833	1.258	0.000
	3.98	212.001	213.010	[M-H]-	6	RP			15.114	1.078	0.000
65	0.65	254.982	255.989	[M-H]-	1	HILIC	C_6_H_8_O_9_S	Ascorbate 2-sulfate	7.435	0.885	0.000
	0.72	254.982	255.989	[M-H]-	1	RP			9.434	0.736	0.000
66	0.65	187.008	188.014	[M-H]-	5	HILIC	C_7_H_8_O_4_S	p-Cresol sulfate	4.667	0.989	0.000
	4.91	187.007	188.014	[M-H]-	0	RP			6.936	0.939	0.002
67	4.95	283.082	284.090	[M-H]-	1	RP	C_13_H_16_O_7_	p-Cresol glucuronide	4.444	0.811	0.006
68	0.69	215.032	216.040	[M-H]-	2	RP	C_5_H_13_O_7_P	2-C-Methyl-D-erythritol 4-phosphate	1.733	1.095	0.009

1 Quasi-molecular ions (*m/z*) in this table have four species, including [M+H]+or [M+Na]+(ES+ mode) and [M−H]−or [M+HCOO]−(ES − mode)

2 Hydrophilic interaction chromatography

3 Reversed-phase chromatography

4 The statistical significance of control, model and fasudil group using one-way ANOVA

### Possible metabolic pathways of differential metabolites

**Figure 5:**
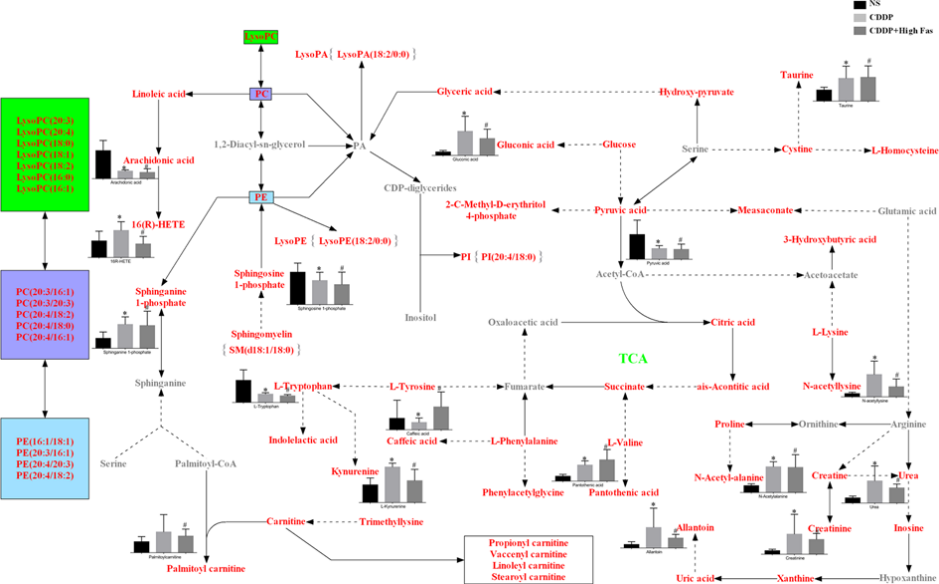
Metabolic pathway network in kidney tissues and the effect of Fas on CDDP-induced kidney injury The levels of related differential metabolites identified in the NS, CDDP, and CDDP+ High Fas Groups are showed in the histogram. The different metabolites in the colored square frame refer to the same species of LysoPC, PC, and PE. The red squares represent metabolites found in the study, whereas the gray squares represent metabolites not found in the study, which were predictable metabolites in the pathway network. The solid arrows indicate the direct production of downstream or upstream metabolites and dotted arrows indicated the indirect production of downstream or upstream metabolites, * means statistic differences (*P* < 0.05) between CDDP Group versus NS Group, # means statistic differences (*P* < 0.05) between CDD+ High Fas Group versus CDDP Group.

According to the network databases MetaboAnalyst (http://www.metaboanalyst.ca) and KEGG (http://www.genome.jp/kegg), the 68 differential metabolites were constructed into a metabolic-pathway map (Figure 5). The tricarboxylic acid cycle in the energy metabolism was used as the center in this metabolic map, which involved multiple metabolic pathways that mainly included lipid metabolism and amino-acid metabolism. In terms of lipids, three phospholipids—lysophosphatidylcholine (LysoPC), phosphatidylcholine (PC), and phosphatidylethanolamine (PE)—were the main metabolites of the pathway. In the amino-acid metabolism, the metabolism of three aromatic amino acids—tryptophan, tyrosine, and phenylalanine—was dominant, which indicates that these metabolism pathways may play key roles in mediating the protective effect of Fas on CDDP-induced kidney injury and this metabolic pathway map may be related to the mechanism underlying the nephrotoxicity caused by CDDP and the renal protection of Fas, Deeper investigation of these pathways should be helpful in elucidating the therapeutic mechanism of Fas.

## Discussion

As a traditional anti-tumor drug, CDDP has been demonstrated to exhibit potent nephrotoxicity. There are four main pathophysiological mechanisms of AKI, namely proximal tubule injury, oxidative stress, inflammation, and renal vascular injury. We successfully constructed a mouse model of CDDP-induced AKI via intraperitoneal injection of CDDP. On this basis, we used Fas as a therapeutic intervention in CDDP-induced AKI mice and demonstrated that Fas had a protective effect on CDDP-induced AKI. Additionally, we used metabolomeic methods to analyze the metabolites in mouse blood samples and searched for potential biomarkers of AKI at the level of endogenous metabolites, attempting to elucidate the mechanisms of CDDP-induced AKI and Fas-mediated renal protection.

Among the 68 differential metabolites found, there were 19 phospholipids, of which PC, LysoPC, and PE were dominant. The fold change of PC in the CDDP group was less than 1 relative to the control group, while the fold change of PC in the Fas group was more than 1 relative to the CDDP group. These findings indicated that the PC level in the mouse blood decreased after the injection of CDDP but increased in mouse blood after the administration of Fas. It has been reported in the literature that PC has a protective effect on CDDP-induced nephrotoxicity in rats [31]. Similarly, in the study, the PC level was substantially reduced after injection of CDDP in mice, which may be due to PC reducing the degree of CDDP-induced kidney damage through its antioxidative activity [32] and a large amount of PC being consumed. In the blood of mice given Fas, PC was significantly higher than that in mice with CDDP-induced AKI that were not given Fas, suggesting that Fas has a protective effect on CDDP-induced AKI, reduces the oxidative stress induced by CDDP, and prevents excessive consumption of PC. As a metabolite of PC, the LPC level was also reduced in the CDDP group, but the difference between the Fas group and the CDDP group was not significant. LPC participates in inflammatory responses through various pathways in the body [33], thereby affecting CDDP-induced AKI. Because a large amount of PC is used to reduce the damage caused by CDDP in the body, a greater amount of PE needs to be converted to PC. Therefore, the PE level in the CDDP group was significantly higher than that in the control group, while the PE level in the Fas group was higher than that in the CDDP group. We hypothesize that Fas may reduce tissue damage from CDDP by increasing PE levels. Sphinganine-1-phosphate is also a metabolite of PE, and its content in the blood of the CDDP group also showed an upward trend. LysoPA (18:2/0:0) can be produced by LysoPE (18:2/0:0), and it has a great influence on neutrophil chemotaxis. In the present study, the LysoPA blood level during CDDP-induced AKI was decreased. In addition, phosphatidylinositol (PI) (20:4/18:0), sphingomyelin (SM; d18:1/18:0), and their products are related to signal transduction. The use of CDDP can increase the blood contents of these molecules, suggesting that CDDP may induce related signaling transductions in the body to become abnormal. Sphingosine 1-phosphate is also a metabolite of SM (d18:1/18:0). Sphingosine 1-phosphate can increase the level of prostaglandin E2 (PGE2) by inducing the expression of cyclooxygenase-2 (COX-2) [34], thereby affecting inflammatory responses in the of kidney. Additionally, studies have shown that sphingosine 1-phosphate can protect the kidney with AKI by activating sphingosine 1-phosphate receptors [35], and the magnitude of this protective effect was reduced when the sphingosine 1-phosphate levels in the CDDP group were decreased. There are also reports indicating that sphingosine 1-phosphate can activate Rho-kinase [36], which can damage renal tubules [16]. The body reduces the level of Rho-kinase-induced damage to the renal tubules by reducing the level of sphingosine 1-phosphate, and Fas protects the kidneys because it is a Rho-kinase inhibitor. In the present study, the level of sphingosine 1-phosphate in the Fas group was lower than that in the CDDP group. We hypothesize that Fas inhibits Rho-kinase by reducing the level of sphingosine 1-phosphate. Unsaturated fatty acids are another type of important PC metabolite. In the present study, we found arachidonic acid. Arachidonic acid can be produced from linoleic acid. Arachidonic acid can directly and indirectly mediate inflammatory response and it is a precursor of prostaglandins, thromboxane, and leukotriene. Arachidonic acid can also generate hydroxyeicosatetraenoic acid (HETE) under the action of cytochrome P450 (CYP450), and we detected 16R-HETE in the present study, which can inhibit the adhesion and aggregation of neutrophils. HETE regulates disease processes by affecting intravascular balance, inflammation, cell growth, apoptosis, and oxidative stress [37]. The most important mechanisms underlying kidney injury induced by CDDP are inflammation and oxidative stress. In the present study, the level of 16R-HETE was considerably elevated in the blood of mice injected with CDDP, and the body inhibited inflammatory responses and oxidative stress by increasing 16R-HETE levels.

In the present study, we found that the levels of eight amino acids showed dramatic differences in the mouse sera among the three groups, including six essential amino acids (L-valine, L-lysine, L-proline, L-tryptophan, L-phenylalanine, and L-tyrosine) and two non-essential amino acids (L-homocysteine and L-kynurenine). L-proline, L-tryptophan, L-phenylalanine, and L-tyrosine showed similar trends in the mice from all three groups, and their concentrations were lower in the CDDP group than in the control group. The trends of L-valine, L-lysine, L-homocysteine, and L-kynurenine were opposite; their concentrations in the CDDP group were higher than those in the control group, and their concentrations in the Fas group were lower than those in the CDDP group. It has been shown in the literature that L-tryptophan can inhibit the early stage of renal failure caused by adriamycin, [38], so a large amount of L-tryptophan may have been consumed in the mice from the CDDP group in our present study. It can be seen on the metabolic pathways map that L-tyrosine can be converted to L-tryptophan. Among the differential metabolites found in the present study, Indolelactic acid and L-kynurenine were the products of L-tryptophan. The receptors of tyrosine kinases (Eph family) serve as key modulators of various cellular functions [39]: it has been reported that overexpression of EphA1 in the kidney attenuates renal fibrosis and improves renal function by inhibiting Rho and MAPK [40]. As the inhibitor of Rho kinases, Fas may alleviate kidney injury by promoting the expression of EphA1, which in turn activates tyrosine kinases. Higher activation of tyrosine kinases consumes more tyrosine, which may indicate why the level of tyrosine was lower after the administration of Fas than in the CDDP group in our present study. Among the metabolites of L-phenylalanine, caffeic acid and phenylacetylglycine were the differential metabolites found in the present study. Caffeic acid phenethyl ester has been shown to have protective effects against nephrotoxicity and oxidative kidney damage. [41]. The decrease in the level of caffeic acid in the present study may have been due to the synthesis of caffeic acid phenethyl ester to attenuate CDDP-induced nephrotoxicity. It has also been reported that caffeic acid inhibits organic anion transporters (OAT1 and OAT3) in rat kidneys. [42] However, there has been no report on the relationship between caffeic acid and organic cation transporter (OCT), and OCTs have a close relationship with the entry of CDDP into kidney cells [43]. Phenylacetylglycine may be a suitable biomarker associated with mitochondrial toxicity [44]. Pantothenic acid is produced by L-valine metabolism, and it is the raw material of coenzyme A (CoA). In the present study, the pantothenic acid level of the model group showed an upward trend compared with that of the control group, which may indicate that CDDP causes abnormal energy metabolism. N-acetyllysine and N-acetylalanine were produced by L-lysine and L-proline, respectively, and their levels were increased significantly in the CDDP group in the present study. N-acetyllysine is associated with histone acetylation. Low-dose histone deacetylase inhibitors can protect cells *in vitro* and attenuate CDDP-induced apoptosis. [45]. Both L-homocysteine and taurine are produced by cysteine. Among the differential metabolites found in the present study, L-homocysteine and taurine exhibited similar trends. Specifically, the L-homocysteine and taurine levels in the CDDP group showed an upward trend relative to those in the control group, and the levels in the Fas group were lower than those in the CDDP group. L-homocysteine and taurine have antioxidative effects in the body, and studies have shown that taurine can exert anti-inflammatory effects through its antioxidative effects to attenuate CDDP-induced nephrotoxicity. [46]. Urea is the main final catabolic product of amino acids, and the fold change of the CDDP group relative to the control group was 3.544 in the present study, while the fold change of the Fas group relative to the CDDP group was 0.796. Creatine is a metabolite of arginine and it is dehydrated to form creatinine. The fold change of the creatinine level in the CDDP group relative to the control group was 5.343 in the present study, while the corresponding fold change in the Fas group relative to the CDDP group was 0.731. Because urea and creatinine are mainly excreted through the urine, an increase in their blood levels may suggest damaged kidney function, which may have confirmed that the CDDP-induced kidney injury model was successfully established in our present study. Creatinine and urea nitrogen are also important indicators for evaluating kidney function. Collectively, these results demonstrate that Fas had a protective effect on kidney function in the present study.

Energy metabolism also showed dramatic changes in the present study. It is based on the tricarboxylic acid cycle and involves the energy metabolism of sugars, amino acids, and fatty acids. Citric acid, cis-aconitic acid, and succinic acid are three organic acids in the tricarboxylic acid cycle. In the present study, their blood levels in the mice from the three groups showed substantial changes, but their trends were not all consistent. The blood glucose level in the CDDP group was significantly higher than that in the control group, and the concentration of pyruvic acid—the main product of glucose—was considerably lower in the CDDP group in the present study. The concentration of gluconic acid, the other product of glucose, was increased by more than seven times in the CDDP group, which may be due to the pathway by which pyruvic acid is produced from glucose being blocked. The serum levels of the other two non-primary products of pyruvic acid—mesaconic acid and 2-C-methyl-D-erythritol 4-phosphate—did increase in the CDDP group, suggesting that the normal energy metabolism pathway had been inhibited. The increased levels of mesaconic acid and 2-C-methyl-D-erythritol 4-phosphate may also be associated with a decrease in pyruvic acid levels in the CDDP group in the present study. Long-chain fatty acids enter mitochondria by forming fatty acyl carnitine. The blood levels of four long-chain fatty acyl carnitines—palmitoylcarnitine, vaccenylcarnitine, linoleylcarnitine, and stearoylcarnitine—found in the present study were significantly higher in the CDDP group, and those of the Fas group were lower than those in the CDDP group. However, the level of propionylcarnitine—another short-chain fatty acyl carnitine—was lower in the mice from the CDDP group, and the short-chain carnitine is produced by β-oxidation of long-chain carnitine. The main source of proximal tubule energy is fatty acids, and CDDP can affect the oxidation of fatty acids by inhibiting kidney PPAR-α, [47], such that the four long-chain fatty acyl carnitines cannot be oxidized and continue to increase. Because short-chain fatty acyl carnitines cannot be normally generated and enter the tricarboxylic acid cycle, the short-chain fatty acyl carnitine, propionylcarnitine, is continuously consumed and reduced. At the same time, Fas may protect fatty acid metabolism in the kidney, thereby reducing the level of CDDP-induced kidney damage. We further explored whether Fas had an agonistic effect on peroxisome proliferator-activated receptor α (PPAR-α). The reason for the decrease in L-carnitine levels in the CDDP group may be due to CDDP hindering the synthesis of L-carnitine, or it may be because fatty acids constantly bind to L-carnitine, which cannot be released via oxidation.

We found inosine, xanthine, uric acid, and allantoin to be metabolites in purine metabolism. A large amount of xanthine dehydrogenase can be converted into xanthine oxidase under conditions of adenosine triphosphate (ATP) deficiency, and inosine can produce hypoxanthine, which is first oxidized to xanthine under the action of xanthine oxidase and then oxidized to uric acid. In this process, the levels of inosine, xanthine, and uric acid were significantly lower in the CDDP group than in the control group in the present study. The decrease in inosine and xanthine levels in the present study may have been be due to an increase in the level of xanthine oxidase. Under the action of CDDP, the body produces a large number of oxygen free radicals and causes oxidative stress responses. Moreover, oxidative stress contributes to the activation Rho-kinase, which causes target organ injury [48]. Uric acid has an antioxidative effect and a large amount of uric acid is oxidized to allantoin, which can be used as a marker of oxidative stress [49]. The level of uric acid is then greatly reduced and the content of allantoin increased. In the present study, the allantoin level in the CDDP group was significantly higher than that in the control group, while the allantoin level in the Fas group was lower than that in the CDDP group. This may be because Fas, an inhibitor of Rho-kinase, can reduce the content of oxygen free radicals caused by CDDP.

We also found several nephrotoxic substances and substances with large fold changes in these differential metabolites. Indoxyl sulfate and p-cresol sulfate are well-defined nephrotoxic substances. Under the action of intestinal flora, tryptophan and tyrosine produce indoxyl and p-cresol, which are transported to the liver to carry out sulfation to form indoxyl sulfate and p-cresol sulfate. We also found p-cresol glucuronide, which is the product of p-cresol glucuronidation. The blood levels of these three substances increased substantially after the injection of CDDP in mice in the present study. The highest fold change (15.114) of the CDDP group compared with that of the control group was observed in the indoxyl sulfate level in the RPLC mode in the present study, and indoxyl sulfate can act as a sensitive indicator to determine chemical-induced AKI. [50]. Indoxyl sulfate and p-cresol sulfate can induce inflammation in proximal tubule cells [51] and indoxyl sulfate can induce apoptosis in glomerular mesangial cells [52]. In the present study, there was no significant decrease in the blood levels of these two substances in the mice injected with Fas, indicating that Fas cannot attenuate CDDP-induced increases in the levels of these substances. The fold change of the CDDP group compared with that in the control group at the aminobenzamide level was 12.142. The increase in the aminobenzamide level enhanced the antioxidative activity [53] and reduced further oxidative damage to the kidney. Ascorbate 2-sulfate also has a strong antioxidative effect, and in RPLC mode, the fold change of the CDDP group was 9.434 compared with the control group.

## Conclusions

In the present study, we analyzed the complex process of CDDP-induced kidney injury from the level of endogenous metabolites by metabolomics and explored the mechanisms underlying the early action of Fas to attenuate CDDP-induced kidney injury. We found 68 potential biomarkers involving phospholipid metabolism, amino-acid metabolism, energy metabolism, and purine metabolism. Additionally, we preliminarily elucidated possible related metabolic pathways and explored the mechanisms of CDDP-induced kidney injury and Fas-mediated renal protection. These findings may provide a reference for early diagnosis and intervention of AKI. However, the molecular mechanisms underlying CDDP-induced kidney injury and Fas-mediated renal protection require further investigation.

## Supplementary Material

Supplementary Figures S1-S2

## Abbreviations

AKI: acute kidney injury
CDDP: Cisplatin
Cys-C: cystatin c
CYR-61: cysteine-rich 61
FGF-23: fibroblast growth factor-23
IGFBP7: insulin-like growth factor binding protein 7
KIM-1: kidney injury molecule-1
NGAL: neutrophil gelatinase associated lipocalin
OCT: organic cation transporter
TIMP-2: tissue inhibitor of metalloproteinase-2
