# Supplementary figures and images for: Metabolomics study of fasudil on cisplatin-induced kidney injury
Source: Biosci Rep. 2019 Nov 19;39(11):BSR20192940. doi: 10.1042/BSR20192940 (PMC6863766; doi:10.1042/BSR20192940)

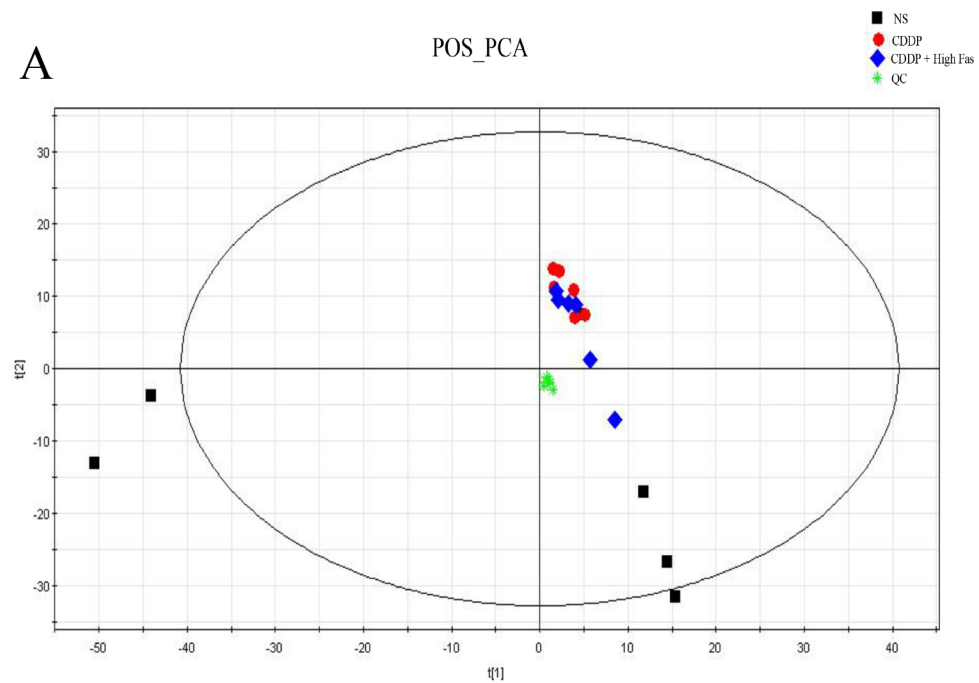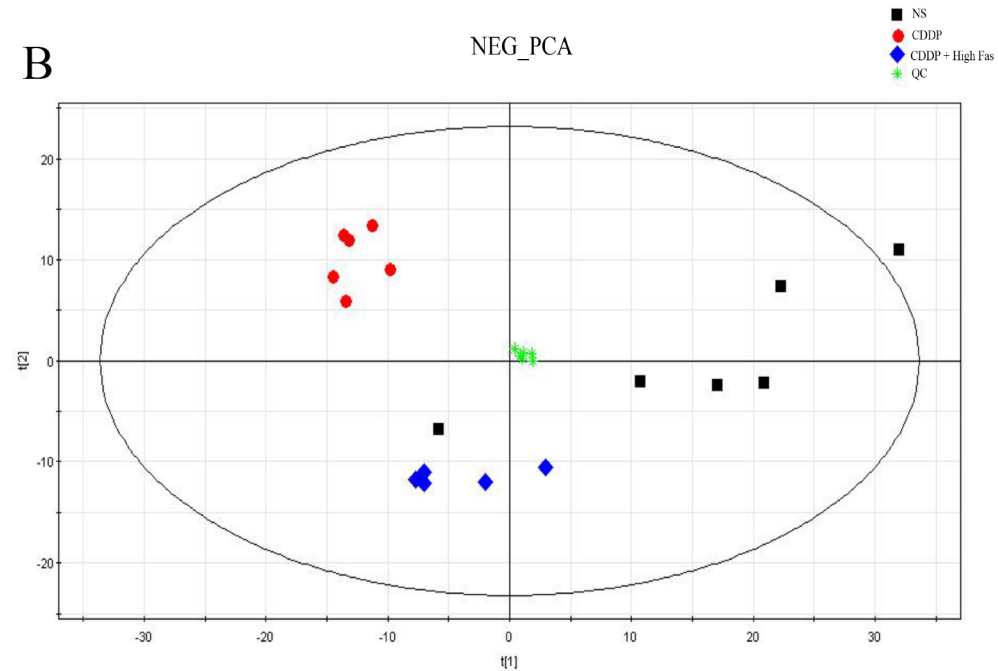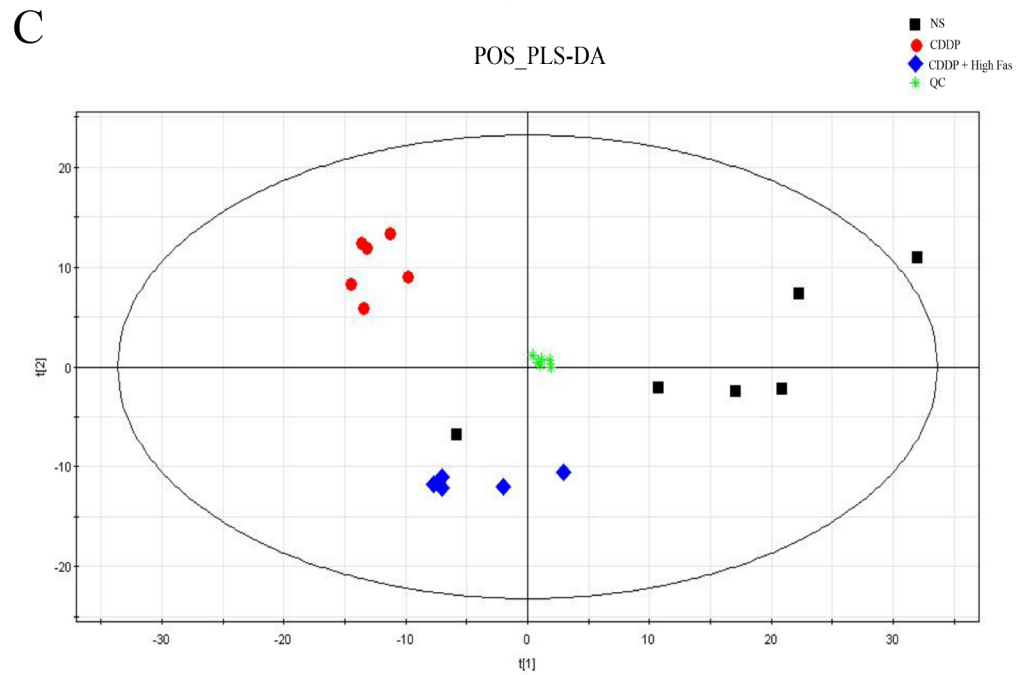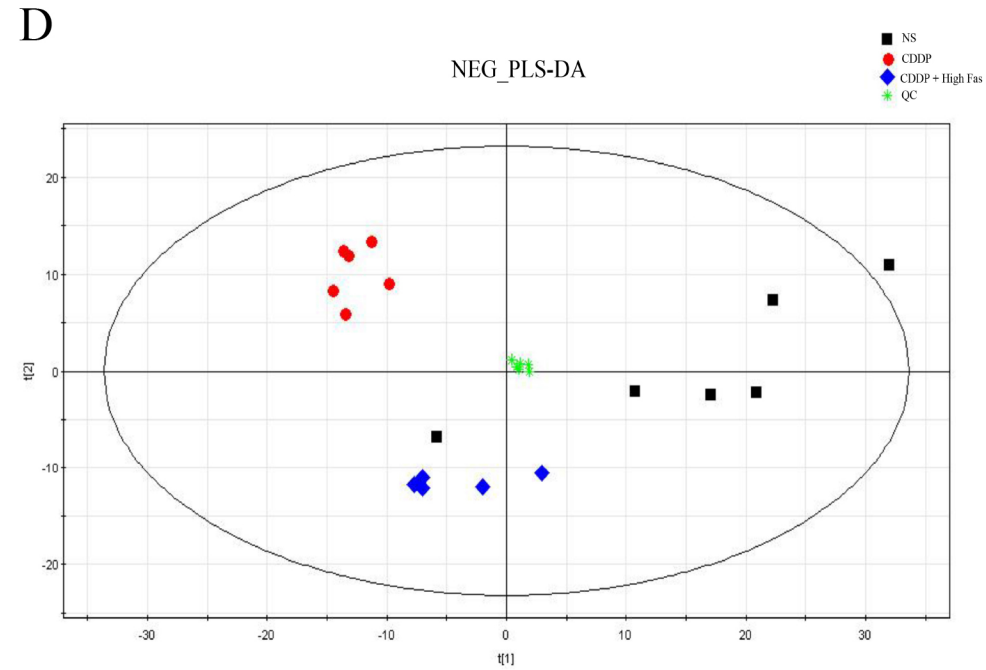

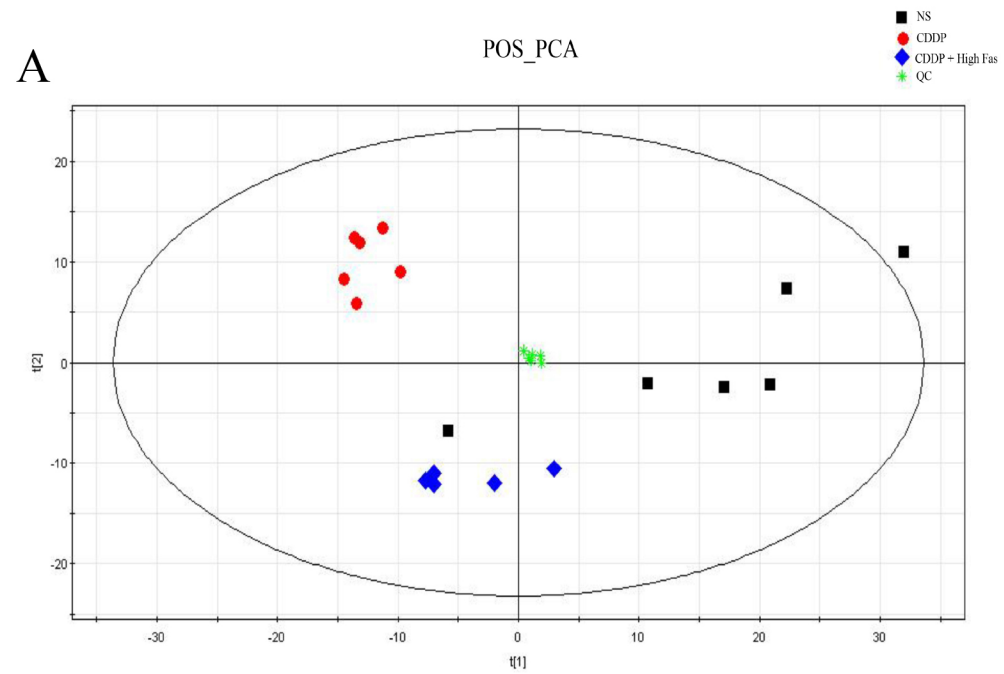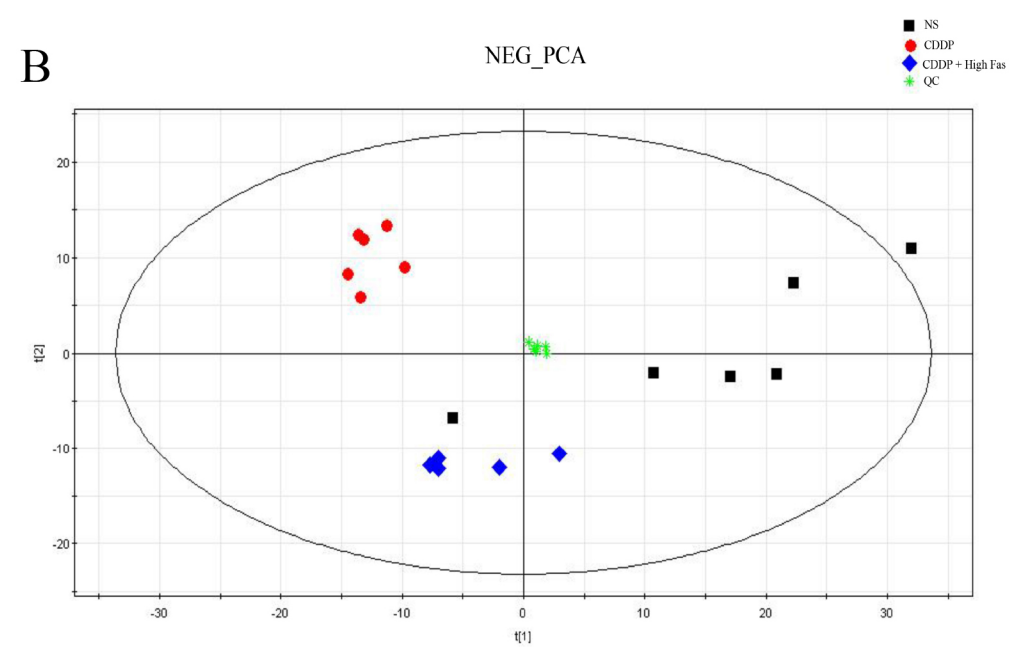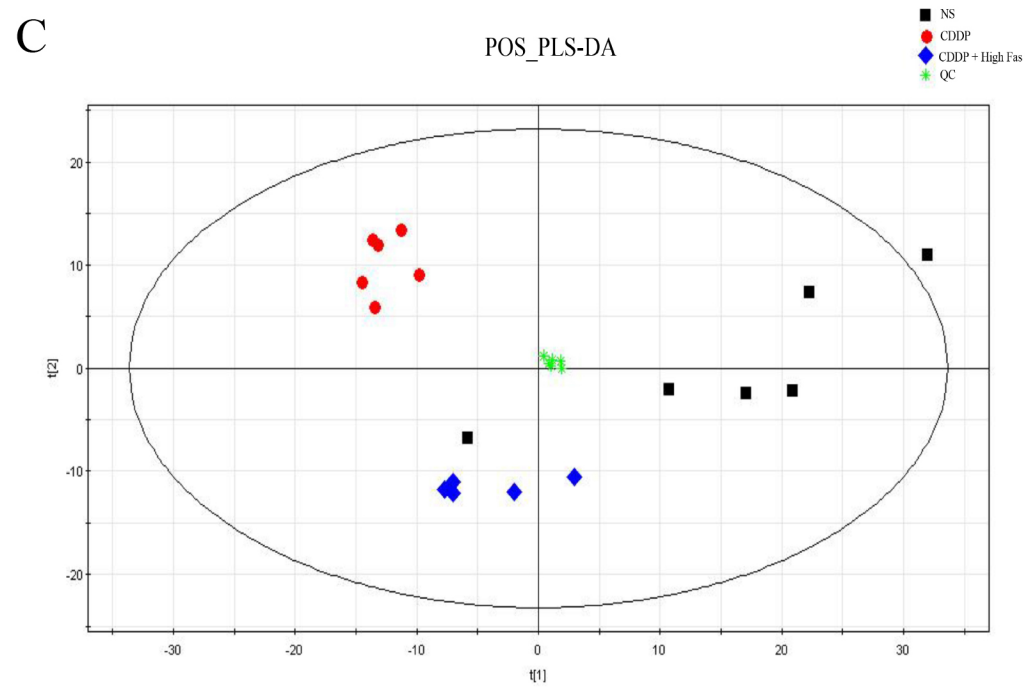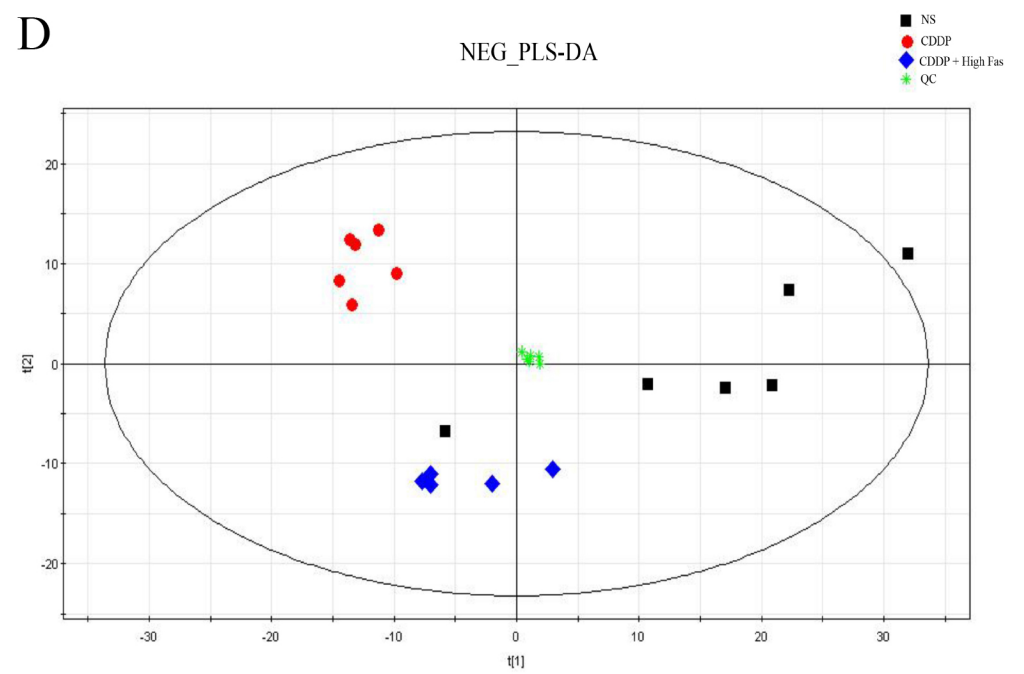

Supplement: Supplementary Figures S1-S2 [file BSR-2019-2940_supp.pdf]
